# Beyond the breast: systemic autoimmune and malignancy associations in idiopathic granulomatous mastitis

**DOI:** 10.1097/JW9.0000000000000276

**Published:** 2026-08-14

**Authors:** Dev Patel, Sach Thakker, Nanette B. Silverberg, Miriam K. Pomeranz

**Affiliations:** a Icahn School of Medicine at Mount Sinai, New York City, New York; b Georgetown University School of Medicine, Washington, DC; c Department of Dermatology and Pediatrics, Icahn School of Medicine at Mount Sinai, New York City, New York; d Ronald O. Perelman Department of Dermatology, New York University School of Medicine, New York City, New York

**Keywords:** autoimmune comorbidities, breast inflammation, granulomatous disease, idiopathic granulomatous mastitis, propensity-score matching, real-world data

**What is known about this subject in regard to women and their families?**

- Idiopathic granulomatous mastitis is a rare inflammatory breast condition that primarily affects women of reproductive age, often during or after pregnancy, and can cause significant physical discomfort, emotional distress, and diagnostic uncertainty for patients and their families.
- The condition frequently mimics infection or breast cancer, leading to anxiety, repeated imaging or biopsies, and prolonged treatment courses, but it has traditionally been viewed as a localized breast disease without clear systemic implications.

**What is new from this article as messages for women and their families?**

- This study suggests that idiopathic granulomatous mastitis may be associated with systemic autoimmune disease and a markedly higher prevalence of breast cancer compared with similar women without the condition.
- These findings highlight the importance of ongoing medical follow-up, attention to autoimmune symptoms, and appropriate breast evaluation, helping women and their families better understand potential health implications beyond localized breast inflammation.

Granulomatous mastitis (GM) is an uncommon inflammatory disorder marked by granulomatous lobulocentric inflammation with macrophage and mixed cell infiltrate.^1^ Although often viewed as localized, evidence suggests systemic immunologic features, including associations with thyroid autoimmunity, connective-tissue disease, and sarcoidosis.^2,3^ Population-level studies assessing autoimmune and oncologic comorbidities are limited. Given overlap between granulomatous inflammation and dysregulated IL-23/IL-17, interferon, and macrophage-driven signaling,^1,4^ we examined autoimmune and malignancy-associated outcomes in adults with GM versus matched controls.

We queried the TriNetX Global Collaborative Network, a de-identified electronic health record network, to identify adults with GM using the International Classification of Diseases, 10th revision N61.20-family codes, excluding infectious mastitis. TriNetX enables propensity-matched comparisons difficult in single-center datasets. Because GM primarily affects parous women,^4^ analyses were restricted to patients with at least 1 prior pregnancy to improve specificity. GM cases were propensity-score matched 1:1 to adults presenting for routine examination (Z00.00) on age, race, ethnicity, granulomatous diseases, prior pregnancies, and baseline autoimmune comorbidities. Matching yielded 4,883 patients per cohort (Table 1). Autoimmune outcomes were assessed beginning 1 day after the index diagnosis. Breast cancer prevalence was evaluated, with colon cancer as a comparator malignancy. TriNetX generated risk differences (RD), risk ratios (RR), and 95% confidence intervals.

**Table 1:** Baseline characteristics in GM cohort vs. controls before and after propensity-score matching

Characteristic	Before PSM, *n* (%)			After PSM (*n* %)		
	GM (n = 4,894)	Control (n = 3,513,876)	SMD	GM (n = 4,883)	Control (n = 4,883)	SMD
Age at index, mean ± SD	41.5 (13.8)	46.3 (18.1)	0.304	41.5 (13.8)	41.8 (13.9)	0.021
Sex (%)						
Female	4,894 (100%)	3,513,876 (100%)	—	4,883 (100%)	4,883 (100%)	—
Race (%)						
White	2,454 (50.2%)	2,406,151 (68.8%)	0.388	2,452 (50.2%)	2,447 (50.1%)	0.002
Black or African American	627 (12.8%)	367,848 (10.5%)	0.071	626 (12.8%)	640 (13.1%)	0.009
Asian	351 (7.2%)	127,634 (3.7%)	0.156	351 (7.2%)	350 (7.2%)	0.001
Native Hawaiian or Other Pacific Islander	43 (0.9%)	11,816 (0.3%)	0.07	42 (0.9%)	51 (1.0%)	0.019
Ethnicity (%)						0.004
Hispanic or Latino	1,542 (31.5%)	192,206 (5.5%)	0.711	1,533 (31.4%)	1,541 (31.6%)	
Comorbidities (%)						
Sarcoidosis	31 (0.6%)	7,702 (0.2%)	0.063	30 (0.6%)	37 (0.8%)	0.017
Tuberculosis	14 (0.3%)	3,523 (0.1%)	0.042	14 (0.3%)	15 (0.3%)	0.004
Granulomatous disorders of skin/subcutis	141 (2.9%)	10,273 (0.3%)	0.208	132 (2.7%)	119 (2.4%)	0.017

GM, Granulomatous Mastitis; PSM, propensity-score matching; SMD, standardized mean difference.

GM patients showed higher rates of autoimmune diseases than matched controls. Systemic lupus erythematous was nearly twice as prevalent (1.0% vs 0.6%; RR = 1.58, *P* = .043), and Sjögren syndrome trended higher (0.6% vs 0.4%; RR = 1.35, *P* = .306). Vitiligo was more frequent (0.8% vs 0.5%; RR = 1.60, *P* = .070), though not significant. Thyroid autoimmunity, rheumatoid arthritis, psoriatic disease, and inflammatory bowel disease occurred at similar frequencies. Breast cancer was more common in GM (6.8% vs 1.3%; RR = 5.39, *P* < .001). A combined endpoint of endometrial, fallopian-tube, and peritoneal cancers showed no increase (1.0% vs 0.9%; RR = 1.19, *P* = .41). Colon cancer rates were comparable (0.9% vs 0.8%; RR = 1.07, *P* = .74) (Table 2).

**Table 2:** Odds of comorbidities in GM vs matched controls (any time after diagnosis)

Outcome	Granulomatous Mastitis cohort (n = 4,883)	Control cohort, (n = 4,883)	Anytime after diagnosis risk (95% CI)		
			Risk difference	Relative risk	*P* value
Crohn’s disease	18 (0.4%)	19 (0.4%)	−0.000 (−0.003 to 0.002)	0.947 (0.498-1.803)	.869
Ulcerative colitis	15 (0.3%)	26 (0.5%)	−0.002 (−0.005 to 0.000)	0.577 (0.306-1.088)	.085
Sjögren syndrome	27 (0.6%)	20 (0.4%)	0.001 (−0.001 to 0.004)	1.350 (0.758-2.404)	.306
Systemic lupus erythematosus	49 (1.0%)	31 (0.6%)	0.004 (0.000-0.007)	1.581 (1.010-2.474)	**.043**
Rheumatoid arthritis	48 (1.0%)	42 (0.9%)	0.001 (−0.003 to 0.005)	1.143 (0.757-1.726)	.525
Psoriatic arthritis	13 (0.3%)	14 (0.3%)	−0.000 (−0.002 to 0.002)	0.929 (0.437-1.973)	.847
Psoriasis	53 (1.1%)	64 (1.3%)	−0.002 (−0.007 to 0.002)	0.828 (0.577-1.189)	.306
Graves disease	22 (0.5%)	26 (0.5%)	−0.001 (−0.004 to 0.002)	0.846 (0.480-1.491)	.563
Hashimoto thyroiditis	58 (1.2%)	74 (1.5%)	−0.003 (−0.008 to 0.001)	0.784 (0.557-1.103)	.161
Vitiligo	40 (0.8%)	25 (0.5%)	0.003 (−0.000 to 0.006)	1.60 (0.97-2.64)	.070
Type 1 diabetes mellitus	35 (0.7%)	32 (0.7%)	0.001 (−0.003 to 0.004)	1.094 (0.678-1.764)	.713
Breast cancer	334 (6.8%)	62 (1.3%)	0.056 (0.048-0.063)	5.387 (4.120-7.044)	**<.001**
Endometrial/fallopian-tube/peritoneal cancer (combined)	51 (1.0%)	43 (0.9%)	0.002 (−0.002 to 0.006)	1.19 (0.79-1.78)	.41
Colon cancer	44 (0.9%)	41 (0.8%)	0.001 (−0.003 to 0.004)	1.07 (0.70-1.64)	.74

These results suggest GM may have systemic immune features rather than being solely a localized breast process. The elevated systemic lupus erythematous risk aligns with reports of heterogeneous immune activation, including interferon signaling and macrophage-driven pathways.^1–3^ The strong breast cancer association raises clinical and mechanistic questions; while surveillance bias may contribute, granulomatous inflammation could alter stromal microenvironments and promote neoplastic pathways.^5^ Whether this reflects immunologic vulnerability, hormonal interplay, or misclassification warrants further study. The absence of increases in comparator malignancy rates and the combined gynecologic cancer endpoint suggests this may not represent a broad cancer-predisposition signal.

Limitations include reliance on International Classification of Diseases, 10th Revision coding, lack of histologic confirmation, and missing data on disease severity, hormonal status, lactation, or treatment. Some patients may have been coded as GM before cancer diagnosis, partly explaining the association. Nevertheless, the use of a large real-world network and restriction of data evaluation to include only parous patients strengthens internal validity. Clinicians should maintain a low threshold for autoimmune review of systems and serologic testing (eg, antinuclear antibodies and thyroid function testing when indicated). Prospective studies integrating cytokine profiling, transcriptomics, microbiome analysis, and cancer surveillance are needed to clarify autoimmune and oncologic susceptibility.

## Conflicts of interest

The authors made the following disclosures: M.P. reports serving as an author for UpToDate and as a member of the Scientific Advisory Board for Procter & Gamble. For the remaining authors there is no conflicts of interest.

## Funding

None.

## Study approval

N/A.

## Author contributions

DP: Research design, performance of research, data analysis, and writing of the manuscript. ST: Research design, performance of research, data analysis, and writing of the manuscript. MP: Research design, study supervision, critical revision of the manuscript, and data interpretation. NS: Research design, study supervision, critical revision of the manuscript, and data interpretation.
